# EHMT2 affects microglia polarization and aggravates neuronal damage and inflammatory response via regulating HMOX1

**DOI:** 10.1515/tnsci-2022-0276

**Published:** 2023-07-27

**Authors:** Huaitao Yang, Zhifang Chen, Wenhong Gao

**Affiliations:** Department of Neurosurgery, Jingzhou Hospital Affiliated to Yangtze University, No. 26, Chuyuan Ave, Jingzhou District, Jingzhou, Hubei 434020, P.R. China; Department of Obstetrics and Gynecology, Jingzhou Hospital Affiliated to Yangtze University, Jingzhou, Hubei 434020, P.R. China

**Keywords:** ischemic stroke, euchromatic histone lysine methyltransferase 2, heme oxygenase 1, histone methylation, neuronal damage, inflammation

## Abstract

**Objective:**

This research was designed to ascertain the function of euchromatic histone lysine methyltransferase 2 (EHMT2) in ischemic stroke-induced neuronal damage and inflammatory response and its regulatory mechanism.

**Methods:**

Mouse microglia (BV-2 cells) were induced by oxygen glucose deprivation/reoxygenation (OGD/R) to establish a cellular model, and then co-cultured with HT22 hippocampal neurons. After that, HT22 cell viability and apoptosis were evaluated, followed by the measurement of apoptosis-related factors (B-cell lymphoma-2, Bcl-2 associated X, and cleaved-Caspase 3). Meanwhile, the expression of inducible nitric oxide synthase (M1 microglia polarization marker) and arginase 1 (M2 microglia polarization marker) in BV-2 cells was detected, as well as the levels of inflammatory factors (tumor necrosis factor-α, interleukin [IL]-6, IL-10, IL-1β, and IL-4). Additionally, the expression of EHMT2 and heme oxygenase 1 (HMOX1) in BV-2 cells was assessed by quantitative reverse transcription polymerase chain reaction and western blot, and the binding between EHMT2 and HMOX1 was predicted and verified.

**Results:**

OGD/R treatment led to decreased cell viability and increased cell apoptosis in HT22 cells, and aggravated inflammatory response in BV-2 cells. In OGD/R-induced BV-2 cells, EHMT2 and HMOX1 were increasingly expressed, and knockdown of EHMT2 or HMOX1 in BV-2 cells could inhibit neuronal damage and inflammatory response. Moreover, EHMT2 promoted HMOX1 transcription level by histone methylation.

**Conclusion:**

Collected evidence showed that down-regulation of EHMT2 relieved neuronal damage and inflammatory response by inhibiting HMOX1 expression.

## 1 Introduction

Stroke is a multifactorial disease caused by a combination of environmental and genetic factors, and it is a major cause of serious long-term disability and death [1]. It can be classified into ischemic or hemorrhagic, in which the majority of stroke cases are ischemic [2], and ischemic stroke is caused by reduction of blood supply to the brain due to obstruction of a blood vessel [3]. Clinically, thrombolytic therapy is currently the most effective treatment for ischemic stroke [4]. However, reperfusion itself may paradoxically fuel the ischemic injury. Therefore, there is an ongoing urgency to figure out the molecular mechanism for broadening therapeutic strategies in ischemic stroke.

Epigenetic mechanisms (including post-translational histone modification, DNA methylation, and small noncoding RNAs) play vital roles in human neurological disorders [5]. Euchromatic histone lysine methyltransferase 2 (EHMT2), also known as the histone methyltransferase G9a, is considered to be a gene repressor by catalyzing the dimethylation of histone H3 lysine 9 (H3K9) [6]. Recent reports have associated EHMT2 with neuronal functions, including axon growth [7], synaptic plasticity [8], and neuronal differentiation [9]. A previous study demonstrated that repression of EHMT2 expression reduced hippocampal microglia activation and depressive-like behavior [10]. Importantly, EHMT2 was measured to be overexpressed in penumbra neurons and astrocytes after photothrombotic stroke (PTS), and the addition of EHMT2 inhibitor protected penumbra cells from apoptosis and reduced the volume of PTS-induced cerebral infarction [11]. Nevertheless, the contribution of EHMT2 to ischemic stroke is rarely studied.

Heme oxygenase (HMOX) is an important homeostatic microsomal enzyme in vascular biology and cell signaling, having two subtypes, HMOX1 and HMOX2 [12]. The HMOX1 is commonly associated with cardiovascular and neurodegenerative diseases including atherosclerosis and ischemic stroke [13,14]. HMOX1 has been reported to be expressed in microglia, and microglial HMOX1 induction after hemorrhagic stroke was positively associated with hematoma size [15]. University of Cingifornia Sisha Cruz (UCSC) website predicted that there is a peak of trimethylation of lysine 4 on histone 3 (H3K4Me3) in HMOX1 promoter region. Moreover, EHMT2 was negatively correlated with the level of nuclear factor erythroid 2-related factor 2 (NRF2) [16], and HMOX1 transcription is mediated by an intertwined circuit where NRF2 plays a crucial role [17,18]. In a previous study, EHMT2 knockdown led to the activation of the protein kinase (PKR)-like ER kinase/NRF2 pathway and HMOX1 upregulation in leukemia stem cells [19]. Therefore, we speculated that EHMT2 regulated H3K4Me3 to affect the expression of HMOX1, thereby involving the occurrence and development of ischemic stroke, which might provide a promising therapeutic target for the treatment of ischemic stroke.

## 2 Materials and methods

### 2.1 Cell culture

Mouse microglia BV-2 (CL-0493; Procell Life Science & Technology Co., Ltd, Wuhan, China) and HT22 hippocampal neurons (BNCC338235; BeNa Culture Collection, Suzhou, China) were cultivated in minimum essential medium supplemented with 10% fetal bovine serum (FBS) and 1% penicillin/streptomycin and Dulbecco’s modified Eagle medium-high glucose (DMEM-H) including 10% FBS, respectively. All cells were maintained at 37°C with 5% CO_2_.

### 2.2 Oxygen glucose deprivation/reoxygenation (OGD/R) cellular model and grouping

According to the previous methods [20,21], BV-2 cells were washed with phosphate buffer saline (PBS) for three times, and then cultured in glucose-free DMEM and placed in a hypoxia incubation chamber filled with gas mixture of 94% N_2_, 5% CO_2_, and 1% O_2_ for 2 h at 37°C. Next, the medium was replaced by high-glucose DMEM and cells were cultured under normoxic conditions for reoxygenation for 12 h. Cells in Control group were cultured in normal-glucose DMEM at 37°C with 5% CO_2_.

The BV cells were randomly grouped as follows: OGD/R group (BV-2 cells were not transfected and only induced by OGD/R), OGD/R + sh-NC group (BV-2 cells were transfected with the negative control of HMOX1 or EHMT2 knockdown plasmid, and then induced by OGD/R), OGD/R + sh-HMOX1 group (BV-2 cells were transfected with HMOX1 knockdown plasmid, and then induced by OGD/R), OGD/R + sh-EHMT2 group (BV-2 cells were transfected with EHMT2 knockdown plasmid, and then induced by OGD/R), sh-NC group (BV-2 cells were transfected with the negative control of EHMT2 knockdown plasmid), sh-EHMT2 group (BV-2 cells were transfected with EHMT2 knockdown plasmid), OGD/R + sh-NC + OE-NC group (BV-2 cells were co-transfected with the negative controls of EHMT2 knockdown and HMOX1 overexpression plasmids, and then induced by OGD/R), OGD/R + sh-EHMT2 + OE-NC group (BV-2 cells were co-transfected with EHMT2 knockdown plasmid and the negative control of HMOX1 overexpression plasmid, and them induced by OGD/R), OGD/R + sh-NC + OE-HMOX1 group (BV-2 cells were co-transfected with the negative control of EHMT2 knockdown plasmid and HMOX1 overexpression plasmid, and them induced by OGD/R), and OGD/R + sh-EHMT2 + OE-HMOX1 group (BV-2 cells were co-transfected with EHMT2 knockdown plasmid and HMOX1 overexpression plasmid, and them induced by OGD/R). When the cell density reached 60–70% confluence, BV-2 cells were transfected with the corresponding plasmids (purchased from GenePharma, Shanghai, China) in accordance with the manufacturer’s instructions of Lipofectamine^TM^ 3000 kit (Invitrogen, Carlsbad, CA, USA) with a transfection concentration of 75 nM. After the transfections, the cells in each group were cultured in an incubator for 1 h before OGD/R modeling described above, and the effectiveness of the transfection process was evaluated by quantitative reverse transcription polymerase chain reaction (qRT-PCR). The sequences of knockdown plasmids and their controls are shown in Table 1.

**Table 1:** Sequences of short hairpin RNAs

Name of primer	Sequences
sh-NC (HMOX1)	5′-ACAAAGACCAGAGTCCCTCACACAG-3′
sh-HMOX1	5′-ACACAAAGACCAGAGTCCCTCACAG-3′
sh-NC (EHMT2)	5′-GGGCGTCAAGAGTGGCCTATGATAT-3′
sh-EHMT2	5′-GGGAGCTGCAGAAGGTGATCCTTAT-3′

### 2.3 Cell co-culture

BV-2 and HT22 cells were co-cultured in 12 mm transwell inserts with 0.4 μm pore size (Corning). BV-2 cells were maintained on the transwell insert and HT22 cells were kept in a 24-well plate for 24 h of co-culture before further experiments.

### 2.4 qRT-PCR

Total RNA was extracted from the treated BV-2 cells with TRIZOL reagent (16096020, Thermo Fisher Scientific, New York, USA). After the RNA samples with the required concentration and purity were diluted to an appropriate concentration, reverse transcription was implemented using the kit (TaKaRa, Tokyo, Japan). Real time PCR was conducted using a LightCycler 480 PCR instrument (Roche, Indianapolis, IN, USA) according to the user manual of a fluorescence quantitative PCR kit (SYBR Green Mix, Roche, Indianapolis, IN, USA) under the following reaction conditions: 35 cycles of pre-denaturation at 95°C for 5 min, denaturation at 95°C for 10 s, annealing at 56°C for 10 s, and extension at 72°C for 20 s. The internal reference was β-actin, and 2^−ΔΔCt^ method was adopted for data analysis (ΔΔCt = experimental group [Ct target gene − Ct internal control] − control group [Ct target gene − Ct internal control]). Each experiment was run in triplicate. Relevant primers were designed by Sangon Biotechnology Co., Ltd (Shanghai, China) (Table 2).

**Table 2:** Primer sequences used in qRT-PCR analysis

Name of primer	Sequences
Hmox1-F	5′-GGAAATCATCCCTTGCACGC-3′
Hmox1-R	5′-TGTTTGAACTTGGTGGGGCT-3′
Ehmt2-F	5′-TTCCTTGTCTCCCCTCCCAG-3′
Ehmt2-R	5′-GACGGTGACAGTGACAGAGG-3′
β-actin-F	5′-GTGACGTTGACATCCGTAAAGA-3′
β-actin-R	5′-GTAACAGTCCGCCTAGAAGCAC-3′

Note: F, forward; R, reverse.

### 2.5 Western blot

BV-2 and HT22 cells were lysed with 4°C precooled radio-immunoprecipitation assay lysis (R0010, Solarbio, China) containing phenylmethanesulfonyl fluoride to obtain total proteins. The protein concentration was measured using a bicinchoninic acid protein quantitative kit (20201ES76, Yeasen Biotechnology Co., Ltd, Shanghai, China). After sodium dodecyl sulfate (SDS) polyacrylamide gel electrophoresis was performed, the protein was transferred onto a polyvinylidene fluoride (PVDF) membrane, followed by treatment with 5% bovine serum albumin (BSA) for 1 h to block non-specific binding. Diluted primary antibodies against HMOX1 (ab52947, 1:2,000; Abcam, Cambridge, UK), EHMT2 (ab185050, 1:500; Abcam), B-cell lymphoma-2 (Bcl-2) (ab182858, 1:2,000; Abcam), Bcl-2 associated X (Bax) (ab32503, 1:500; Abcam), cleaved-Caspase 3 (ab214430, 1:5,000; Abcam), inducible nitric oxide synthase (iNOS) (ab178945, 1:1,000; Abcam), arginase 1 (Arg1) (ab124917, 1:1,000; Abcam), and β-actin (ab8227, 1:2,000; Abcam) were supplemented onto the PVDF membrane at 4°C for overnight incubation. After Tris-buffered saline with Tween 20 washing, the secondary antibody goat anti-rabbit immunoglobulin G (IgG) (ab6721, 1:5,000; Abcam) tagged with horseradish peroxidase (HRP) was added for 1 h incubation, followed by treatment with electrogenerated chemiluminescence developer (Thermo Fisher, USA) for color development. Finally, Bio-Rad image analysis system (BIO-RAD, USA) was used for photographing and Quantity One v4.6.2 software was used for analysis with gray values of corresponding protein bands/gray value of β-actin protein bands as relative protein content. Each experiment was run in triplicate.

### 2.6 3-(4,5)-Dimethylthiahiazo (-*z*-y1)-3,5-di-phenytetrazoliumromide (MTT)

Logarithmic phase HT22 cells were collected and counted, and cell suspension concentration was adjusted so that the cell density in the 96-well plates was about 5 × 10^4^ cell/well. After grouping, the plate was added with corresponding reagent and replaced in 37°C and 5% CO_2_ incubator for 24–36 h. After that, 50 µL of MTT solution was added and cultured at 37°C for 4 h to reduce MTT to formazan. Then, the plate was taken with the supernatant removed, and each well was dropped with 150 µL dimethyl sulfoxide prior to 10 min low speed oscillation. After the formazan crystal was completely dissolved, the optical density (OD) value of each well at a wavelength of 570 nm was tested. Experiments were independently performed three times. The normalization methods were as follows: the OD value in the Control group was taken as the normal cell viability. Then, the OD value in the OGD/R group was compared to that in the Control group to obtain the ratio that indicated the cell viability after OGD/R treatment. By analogy, the OD value of the OGD/R group was used as a reference in subsequent relevant experiments to observe the changes in cell viability under other different treatments.

### 2.7 Terminal deoxynucleotidyl transferase-mediated dUTP nick-end labeling (TUNEL) assay

The collected HT22 cells were fixed in 4% paraformaldehyde for 30 min, and then washed twice with PBS with the supernatant removed. Next, the cells were treated with 70% cold ethanol for 15 min, followed by PBS washing and the removal of the supernatant. After 5 min incubation with PBS (containing 0.3% Triton X-100), the cells were incubated with TUNEL detection solution (Beyotime, Shanghai, China) for 60 min at 37°C in the dark, and then washed with PBS three times. After fixation with anti-fluorescence quenching solution, the cells were observed under a fluorescence microscope. The nuclei were stained with 4′,6-diamidino-2-phenylindole (DAPI). The nuclei of apoptotic cells showed green fluorescence under the fluorescence microscope, while the nuclei of normal cells stained by DAPI showed blue fluorescence. Cell counting was performed by researchers who were blind to the grouping or specific treatment.

### 2.8 Enzyme linked immunosorbent assay (ELISA)

The levels of inflammatory factors like tumor necrosis factor-α (TNF-α), interleukin (IL)-6, IL-10, IL-1β, and IL-4 were determined using ELISA kits (BD Biosciences, Yansheng Biotechnology Co., Ltd, Shanghai, China). Briefly, the samples were added to a microplate and wrapped at room temperature overnight. After the culture solution was abandoned, the sample was washed three times with PBS for 5 min each. Later, 5% BSA blocking liquid was added (100 μL/well) for 1 h, followed by the addition of primary antibody diluted by PBS (containing 5% BSA) to a 96-well plate with 100 μL per well. After 3 h incubation, the plate was washed three times with PBS for 5 min each time. Then the plate was added with HRP-labeled secondary antibody diluted in PBS (containing 5% BSA). Finally, 10 μL of substrate was added and cultured with the supernatant at 37°C for 10–15 min, and the absorbance value of each sample was measured at 450 nm. Each experiment was repeated three times.

### 2.9 Chromatin immunoprecipitation (ChIP) assay

In accordance with the instructions of an EZ ChIP kit (Millipore, Germany), transfected BV-2 cells were fixed with 1% formaldehyde to crosslink DNA–protein complexes for 10 min. After that, the crosslinking was terminated by adding glycine for 5 min. The cells (10^6^) were lysed with 100 µL SDS lysis buffer (SDS lysis buffer + proteinase inhibitor cocktail II). Next, the DNA from the cell lysate was ultrasonically broken into 200–1,000 bp fragments. IgG antibody (Millipore), EHMT2 monoclonal antibody (ab185050; Abcam), and H3K4Me3 monoclonal antibody (ab213224; Abcam) were used for ChIP assay. The purified DNA products obtained were detected by qRT-PCR to confirm whether the DNA precipitated by antibodies contained the DNA sequence of the target gene and its relative content.

### 2.10 Statistical analysis

Statistical analysis of the collected original data was conducted using GraphPad 9.0 software. All quantitative data were shown as mean ±  standard deviation. *T*-test and one-way analysis of variance were used for comparisons between two groups and among the groups, respectively. Tukey’s multiple comparisons test was used for *post hoc* analysis. A *P* value <0.05 was accepted as statistically significant.

## 3 Results

### 3.1 HMOX1 was highly expressed in OGD/R-induced BV-2 cells

**Figure 1:**
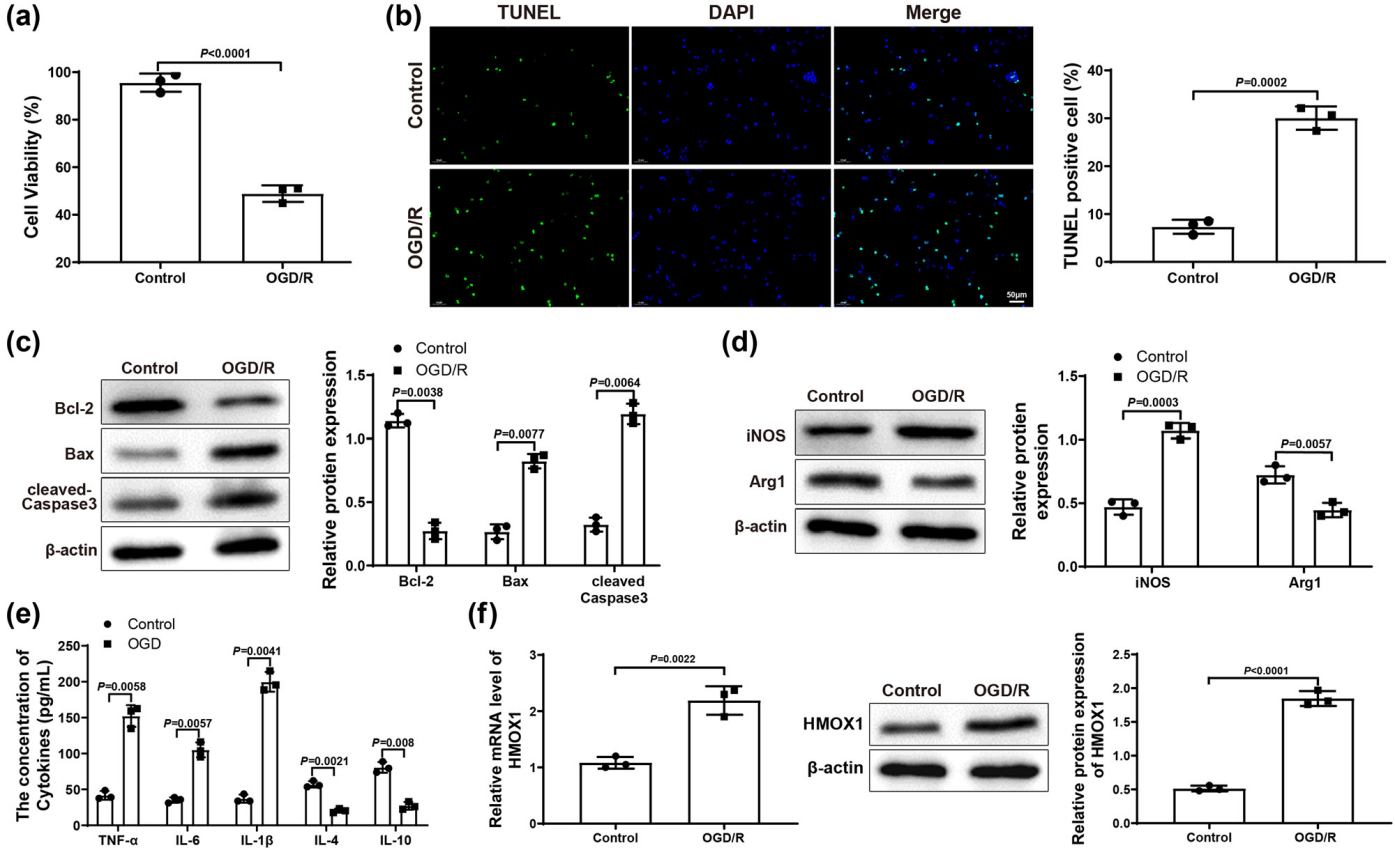
HMOX1 expressed at a high level in OGD/R-induced BV-2 cells. (a) MTT assay was applied to test HT22 cell viability. (b) HT22 cell apoptosis was measured by TUNEL staining. (c) Expression of apoptosis-related proteins (Bcl-2, Bax, and cleaved-Caspase 3) in HT22 cells was evaluated by western blot. (d) Expression of iNOS (a maker for M1 microglia polarization) and Arg1 (a maker for M2 microglia polarization) in BV-2 cells was examined by western blot. (e) Expression levels of inflammatory factors in BV-2 cells were detected by ELISA. (f) mRNA and protein expression of HMOX1 in BV-2 cells was measured by qRT-PCR and western blot. Each experiment was repeated three times. *T*-test was used for comparisons between two groups. OGD/R, oxygen glucose deprivation/reoxygenation.

In order to explore the mechanism of HMOX1 on ischemic stroke, we used OGD/R to induce activation of mouse microglia (BV-2 cells) to simulate ischemic stroke *in vitro*, followed by the exploration of BV-2 cell biological function changes and detection of the damage of mouse hippocampal neurons HT22 cells. Results from MTT assay revealed that OGD/R induction led to markedly decreased HT22 cell viability (Figure 1a). TUNEL staining showed that HT22 cell apoptosis in OGD/R group was higher than that in the Control group (Figure 1b). Additionally, western blots on HT22 cells revealed that OGD/R group had lower expression of Bcl-2 and higher expression of Bax and cleaved-Caspase 3 than that in the Control group (Figure 1c). These results suggested that the HT22 neurons were damaged after co-culture with OGD/R-treated BV-2 cells.

Moreover, we also tested the expression of iNOS (a marker for pro-inflammatory M1 microglia polarization) and Arg1 (a marker for anti-inflammatory M2 microglia polarization) in BV-2 cells. Results from western blot reflected that iNOS increased but Arg1 decreased in OGD/R group (vs Control group; Figure 1d), suggesting that OGD/R treatment induced M1 polarization of BV-2 cells. Meanwhile, ELISA suggested that, in OGD/R group, the levels of pro-inflammatory factors TNF-α, IL-6, and IL-1β were observably enhanced, while the levels of anti-inflammatory factors IL-10 and IL-4 were signally reduced (vs Control group; Figure 1e). Finally, qRT-PCR and western blot results manifested that the expression of HMOX1 in OGD/R group was dramatically elevated compared with Control group (Figure 1f). Above data demonstrated that OGD/R could cause inflammatory response in BV-2 cells, which led to neuronal damage. Additionally, the upregulation of HMOX1 in BV cells suggested that HMOX1 may be involved in the occurrence and development of ischemic stroke.

### 3.2 Knockdown of HMOX1 repressed neuronal damage and inflammatory response

**Figure 2:**
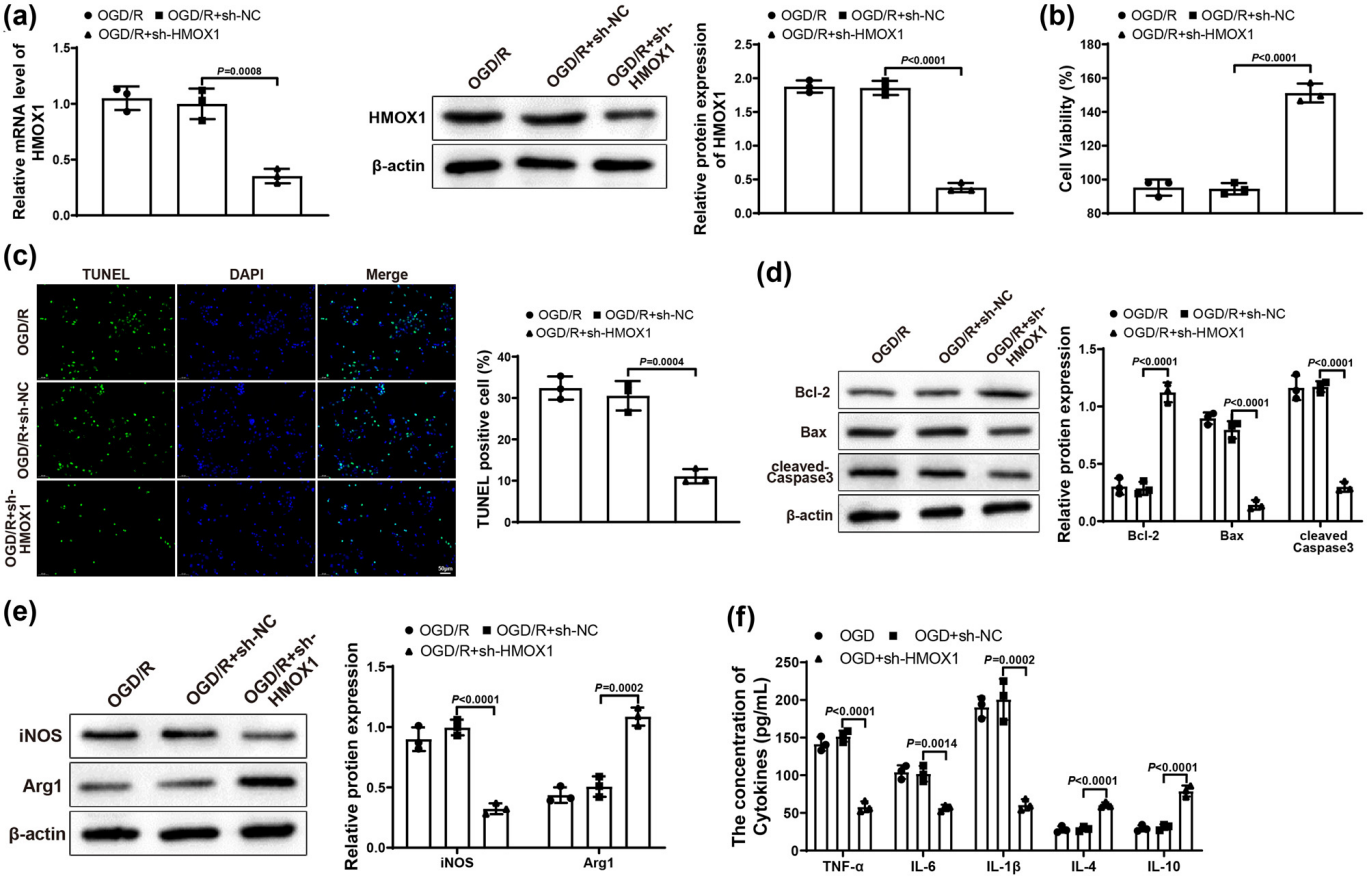
Downregulation of HMOX1 suppressed neuronal damage and inflammatory response. (a) Expression of HMOX1 in BV-2 cells was measured by qRT-PCR and western blot. (b) MTT assay was used to test HT22 cell viability. (c) HT22 cell apoptosis was measured by TUNEL staining. (d) Expression of Bcl-2, Bax, and cleaved-Caspase 3 was evaluated by western blot in HT22 cells. (e) Expression of iNOS and Arg1 in BV-2 cells was examined by western blot. (f) Expression levels of inflammatory factors in BV-2 cells were detected by ELISA. Each assay was performed three times. One-way analysis of variance test was used for comparisons among multiple groups with Tukey’s multiple comparisons test for *post hoc* tests. OGD/R, oxygen glucose deprivation/reoxygenation.

Subsequently, BV-2 cells in Control and OGD/R groups were transfected with sh-NC and sh-HMOX1. Reflected in qRT-PCR or western blot results, OGD/R + sh-HMOX1 group had lower expression of HMOX1 than that in OGD/R and OGD/R + sh-NC groups (Figure 2a), indicating that the transfection efficiency was good. The transfection result of BV-2 cells detected by qRT-PCR in Control group was similar to that of OGD/R group (Figure S1a). MTT assay suggested that OGD/R + sh-HMOX1 group had higher HT22 cell viability than OGD/R and OGD/R + sh-NC groups (Figure 2b). As expected, HT22 cell apoptosis rate in OGD/R + sh-HMOX1 group was clearly decreased compared with OGD/R and OGD/R + sh-NC groups (Figure 2c). Also, the expression of Bcl-2 was obviously elevated and the expression of Bax and cleaved-Caspase 3 was signally repressed in OGD/R + sh-HMOX1 group (vs OGD/R and OGD/R + sh-NC groups; Figure 2d). These results showed that downregulation of HMOX1 in BV-2 cells could relieve neuronal damage in HT22 cells. Compared with OGD/R and OGD/R + sh-NC groups, the expression of iNOS visibly reduced but the expression of Arg1 distinctly increased in OGD/R + sh-HMOX1 group (Figure 2e). In addition, OGD/R + sh-HMOX1 group had lower levels of TNF-α, IL-6, and IL-1β and higher levels of IL-10 and IL-4 (vs OGD/R and OGD/R + sh-NC groups; Figure 2f). Overall, downregulated HMOX1 could alleviate neuronal damage and block inflammatory response.

### 3.3 EHMT2 positively regulated HMOX1 expression through histone methylation

**Figure 3:**
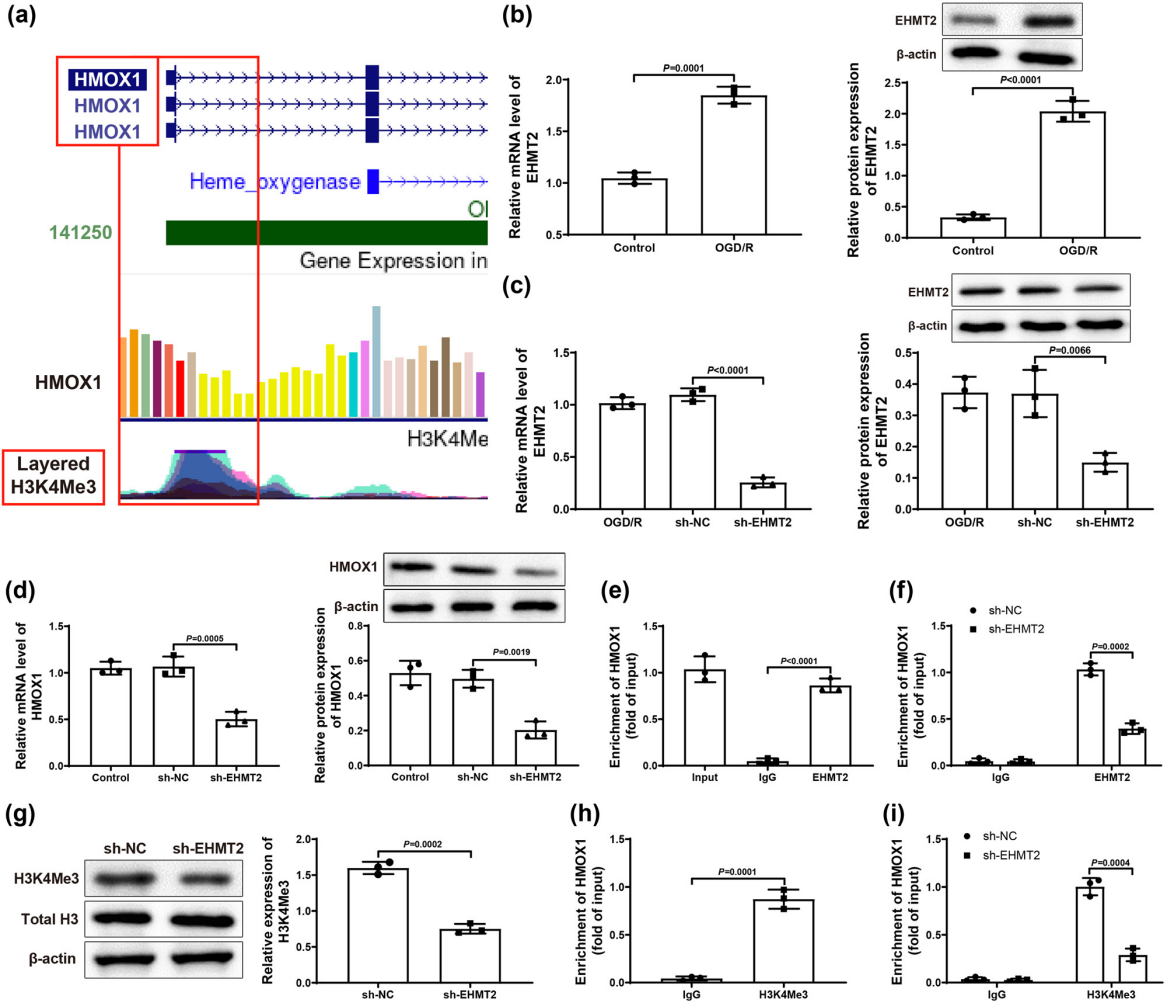
EHMT2 positively modulated HMOX1 expression. (a) UCSC website showed that HMOX1 promoter region had a peak of H3K4Me3. (b) mRNA and protein expression of EHMT2 in BV-2 cells was measured by qRT-PCR and western blot. (c and d) Expression of EHMT2 and HMOX1 was examined by qRT-PCR and western blot. (e) ChIP assay was used to test the binding between EHMT2 and HMOX1 promoter. (f) ChIP assay was used to detect changes in HMOX1 promoter enriched by EHMT2 antibody. (g) Expression of H3K4Me3 was assessed by western blot. (h) ChIP assay was used to measure the H3K4Me3 enrichment in HMOX1 promoter region. (i) Enrichment of H3K4Me3 in HMOX1 promoter region in sh-NC and sh-EHMT2 groups was evaluated by ChIP assay. Three independent experiments were carried out. *T*-test was used for comparisons between two groups.

Through UCSC website (https://genome.ucsc.edu/) analysis, it was found that HMOX1 promoter region had a peak of H3K4Me3 (Figure 3a), suggesting that HMOX1 expression was regulated by H3K4Me3 methylation. Afterward, we hypothesized that EHMT2 might affect HMOX1 expression by modulating H3K4Me3 level, thereby participating in the occurrence and development of ischemic stroke. To confirm this hypothesis, we first used qRT-PCR and western blot to detect the mRNA and protein expression of EHMT2 in BV-2 cells, and the results showed that OGD/R group had higher expression of EHMT2 than in Control group (Figure 3b).

Next, BV2 cells in Control and OGD/R groups were transfected with sh-EHMT2 or sh-NC. Results from qRT-PCR and western blot revealed that the expression of EHMT2 and HMOX1 in sh-EHMT2 group was obviously decreased compared with OGD/R and sh-NC groups, while there was no significant difference between OGD/R group and sh-NC group (Figure 3c and d). The transfection result of BV-2 cells detected by qRT-PCR in Control group was similar to that of OGD/R group (Figure S1b). Later, we used ChIP assay to confirm the binding of EHMT2 and HMOX1 promoter, and the results displayed that the enrichment of EHMT2 in HMOX1 promoter was clearly enhanced compared with IgG group (Figure 3e). In addition, the enrichment of EHMT2 in HMOX1 promoter was markedly decreased in sh-EHMT2 group compared with sh-NC group (Figure 3f). Above results suggested that EHMT2 was enriched in the transcriptional regulatory region of HMOX1 gene.

In order to further explore whether H3K4Me3 was involved in the transcriptional regulation of HMOX1 by EHMT2, we used western blot to explore the expression of H3K4Me3 after transfection of sh-EHMT2. Results manifested that sh-EHMT2 transfection did not change the expression of Total H3, but significantly down-regulated the expression of H3K4Me3 (vs sh-NC group; Figure 3g). Meanwhile, ChIP assay revealed that H3K4Me3 enrichment level in HMOX1 promoter region was dramatically increased (vs IgG group; Figure 3h). In comparison with sh-NC group, the enrichment level of H3K4Me3 in HMOX1 promoter region in sh-EHMT2 group was clearly reduced (Figure 3i). From these results, it was clear that EHMT2 could modulate H3K4Me3 level to regulate HMOX1 expression.

### 3.4 Downregulation of EHMT2 inhibited neuronal damage and inflammatory response

**Figure 4:**
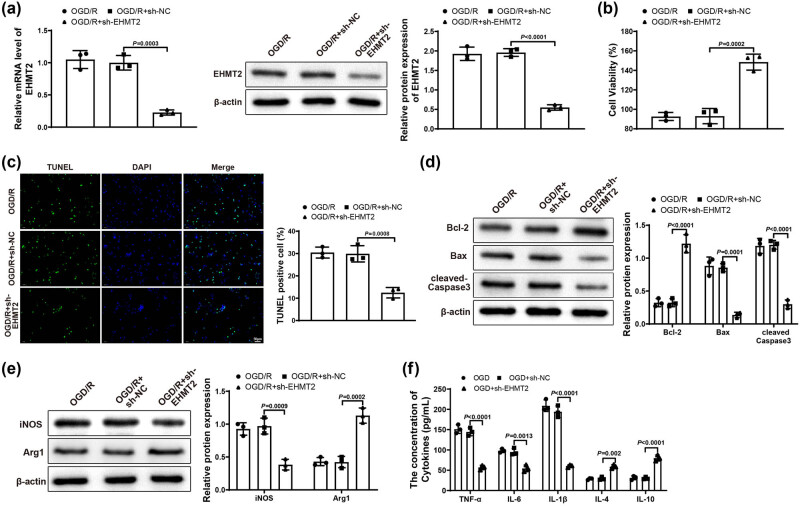
Downregulated EHMT2 repressed neuronal damage and inflammatory response. (a) Expression of EHMT2 in BV-2 cells was tested by qRT-PCR and western blot. (b) MTT assay was applied to test HT22 cell viability. (c) HT22 cell apoptosis was evaluated by TUNEL staining. (d) Expression of Bcl-2, Bax, and cleaved-Caspase 3 in HT22 cells was examined by western blot. (e) Expression of iNOS and Arg1 in BV-2 cells was assessed by western blot. (f) Expression levels of inflammatory factors in BV-2 cells were detected by ELISA assay. Each experiment was repeated three times. One-way analysis of variance test was used for comparisons among multiple groups with Tukey’s multiple comparisons test for *post hoc* tests. OGD/R, oxygen glucose deprivation/reoxygenation.

To further reveal the effect of EHMT2 on ischemic stroke, we transfected BV-2 cells in Control and OGD/R groups with sh-NC and sh-EHMT2. First, qRT-PCR or western blot results reflected that sh-EHMT2 group had lower expression of EHMT2 than OGD/R and OGD/R + sh-NC groups (Figure 4a), showing that the transfection efficiency was good. The transfection result of BV-2 cells detected by qRT-PCR in Control group was similar to that of OGD/R group (Figure S1c). MTT assay showed that OGD/R + sh-EHMT2 group had higher HT22 cell viability than OGD/R and OGD/R + sh-NC groups (Figure 4b). Displayed in Figure 4c, HT22 cell apoptosis rate in OGD/R + sh-EHMT2 group was prominently decreased compared with that in OGD/R and OGD/R + sh-NC groups. In comparison with OGD/R and OGD/R + sh-NC groups, the expression of Bcl-2 was clearly elevated and the expression of Bax and cleaved-Caspase 3 was signally repressed in OGD/R + sh-EHMT2 group (Figure 4d). These results showed that knockdown of EHMT2 could mitigate neuronal damage in HT22 cells. Furthermore, compared with OGD/R and OGD/R + sh-NC groups, iNOS expression appreciably reduced but Arg1 expression noticeably increased in OGD/R + sh-EHMT2 group (Figure 4e). Shown in Figure 4f, OGD/R + sh-EHMT2 group had lower levels of TNF-α, IL-6, and IL-1β and higher levels of IL-10 and IL-4 (vs OGD/R and OGD/R + sh-NC groups). Taken together, knockdown of EHMT2 could reduce neuronal damage and restrain inflammatory response.

### 3.5 Silencing EHMT2 alleviated neuronal damage and inflammatory response by repressing HMOX1 expression

**Figure 5:**
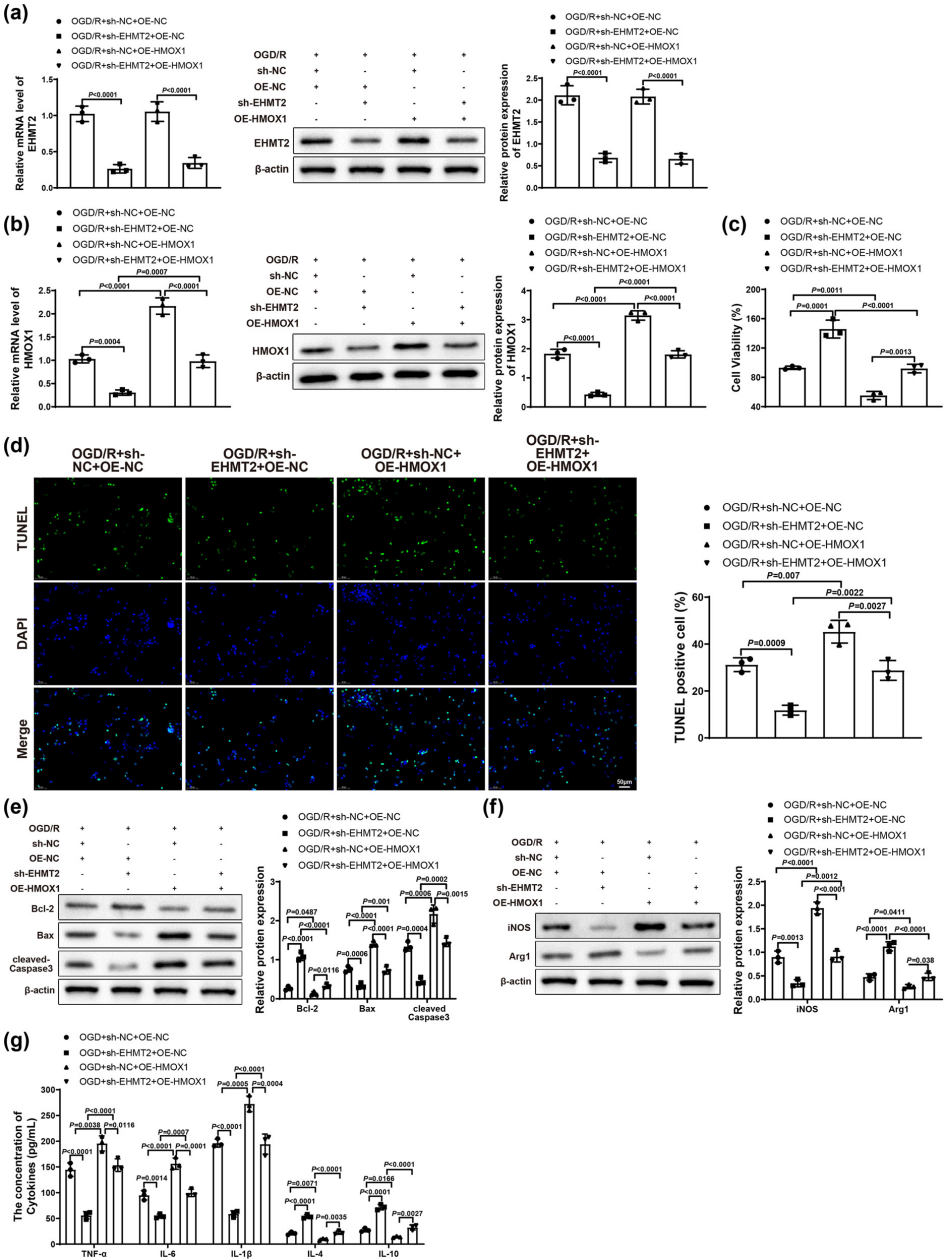
Knockdown of EHMT2 improved neuronal damage and inflammatory response by inhibiting HMOX1 expression. (a and b) Expression of EHMT2 and HMOX1 in BV-2 cells was evaluated by qRT-PCR and western blot. (c) MTT assay was used to detect HT22 cell viability. (d) HT22 cell apoptosis was measured by TUNEL staining. (e) Expression of Bcl-2, Bax, and cleaved-Caspase 3 in HT22 cells was measured by western blot. (f) Expression of iNOS and Arg1 in BV-2 cells was assessed by western blot. (g) Expression levels of inflammatory factors in BV-2 cells were measured by ELISA. Each assay was repeated three times. One-way analysis of variance test was used for comparisons among multiple groups with Tukey’s multiple comparisons test for *post hoc* tests. OGD/R, oxygen glucose deprivation/reoxygenation.

Finally, to further verify the molecular mechanism of EHMT2 affecting ischemic stroke by regulating HMOX1 expression, we co-transfected BV-2 cells in Control and OGD/R groups with sh-NC, sh-EHMT2, OE-NC or OE-HMOX1 plasmids (OGD/R + sh-NC + OE-NC, OGD/R + sh-EHMT2 + OE-NC, OGD/R + sh-NC + OE-HMOX1, and OGD/R + sh-EHMT2 + OE-HMOX1 groups). qRT-PCR or western blot was applied to test the mRNA and protein expression of EHMT2 and HMOX1 in BV-2 cells. Results showed that the expression of EHMT2 was obviously decreased in OGD/R + sh-EHMT2 + OE-NC group versus OGD/R + sh-NC + OE-NC and OGD/R + sh-NC + OE-HMOX1 groups (Figure 5a). Compared with OGD/R + sh-NC + OE-NC and OGD/R + sh-EHMT2 + OE-HMOX1 groups, the expression of HMOX1 was appreciably decreased in OGD/R + sh-EHMT2 + OE-NC group but increased in OGD/R + sh-NC + OE-HMOX1 group (Figure 5b). The transfection result of BV-2 cells detected by qRT-PCR in Control group was similar to that of OGD/R group (Figure S1d). Compared with OGD/R + sh-NC + OE-NC and OGD/R + sh-EHMT2 + OE-HMOX1 groups, HT22 cell viability was markedly enhanced in OGD/R + sh-EHMT2 + OE-NC group but signally reduced in OGD/R + sh-NC + OE-HMOX1 group (Figure 5c). In comparison with OGD/R + sh-NC + OE-NC and OGD/R + sh-EHMT2 + OE-HMOX1 groups, cell apoptosis rate was decreased in OGD/R + sh-EHMT2 + OE-NC group but increased in OGD/R + sh-NC + OE-HMOX1 group (Figure 5d). The expression of Bcl-2, Bax, and cleaved-Caspase 3 was measured by western blot, and results revealed that OGD/R + sh-EHMT2 + OE-NC group had increased Bcl-2 and decreased Bax and cleaved-Caspase 3, while OGD/R + sh-NC + OE-HMOX1 group had reduced Bcl-2 and elevated Bax and cleaved-Caspase 3 (vs OGD/R + sh-NC + OE-NC and OGD/R + sh-EHMT2 + OE-HMOX1 groups; Figure 5e). These results demonstrated that knockdown of EHMT2 could alleviate the damage caused by overexpressed HMOX1 to neuronal cells.

In addition, western blot results showed that, compared with OGD/R + sh-NC + OE-NC and OGD/R + sh-EHMT2 + OE-HMOX1 groups, OGD/R + sh-EHMT2 + OE-NC group had reduced iNOS and increased Arg1, while OGD/R + sh-NC + OE-HMOX1 group had upregulated iNOS and downregulated Arg1 (Figure 5f). As suggested in Figure 5g, OGD/R + sh-EHMT2 + OE-NC group had low levels of TNF-α, IL-6, and IL-1β and high levels of IL-10 and IL-4, while OGD/R + sh-NC + OE-HMOX1 group had upregulated TNF-α, IL-6, and IL-1β and downregulated IL-10 and IL-4 (vs OGD/R + sh-NC + OE-NC and OGD/R + sh-EHMT2 + OE-HMOX1 groups). In summary, silencing EHMT2 could repress HMOX1 expression by inhibiting histone methylation, thus alleviating neuronal damage and blocking cell apoptosis and inflammatory response.

## 4 Discussion

Ischemic stroke is a neurological disorder resulted from loss of blood supply [22]. Dead neurons generate danger-associated molecular patterns, triggering excessive inflammatory responses [23]. Microglia are resident immune cells in the brain and play a central role in post-ischemic inflammation [24]. Microglia can polarize to classic M1 microglia, which release pro-inflammatory cytokines, and M2 microglia, which produce anti-inflammatory cytokines, and the balance of the two counteracting mediators governs the fate of damaged neurons [25]. Thus, development in immunomodulatory approaches will contribute to limit neuronal injury and improve post-stroke functional impairment. Our work demonstrated that knockdown of EHMT2 reduced HMOX1 expression by inhibiting histone methylation, thus alleviating neuronal damage and blocking apoptosis and inflammatory response in hippocampal neurons.

Initially, to explore the specific effect of HMOX1 on ischemic stroke, we first simulated ischemic stroke *in vitro* by OGD/R in microglia (BV-2 cells), and then co-cultured BV-2 cells with HT22 hippocampal neurons. Results from experiments revealed that the HT22 neurons were damaged after co-culture with OGD/R-treated BV-2 cells, and HMOX1 was highly expressed in OGD/R-induced BV-2 cells. Generally, hemoglobin and its degeneration products are deleterious to the brain and heme metabolism can prevent the production of reactive oxygen species and inflammation, which is critical to cerebral recovery [26]. In this regard, up-regulation of HMOX1 may have a protective effect in ischemic stroke. However, sustained up-regulation of the stress protein, HMOX1, in astroglia of the aging and diseased central nervous system is reported as a primary transducer of detrimental stimuli [27]. That may be owing to the beneficial effect of HMOX1 on microglial survival. A previous study showed that middle cerebral artery occlusion upregulated the expression of NRF2 and HMOX1 in the contralesional cerebellar cortex [28]. Another study displayed that high HMOX1 expression promoted microglia survival in the intracerebral hemorrhage environment, further promoting inflammation and oxidative damage [29]. Consistently, in our study, knockdown of HMOX1 alleviated neuronal damage and block inflammatory response, shown by a decreased HT22 cell apoptosis rate, lower levels of TNF-α, IL-6, and IL-1β, and higher levels of IL-10 and IL-4. Moreover, analysis from UCSC website illustrated that there was a peak of H3K4Me3 in HMOX1 promoter region, and subsequent ChIP assay confirmed the binding of EHMT2 and HMOX1 promoter.

Next, we further explored the effect of EHMT2 on ischemic stroke, and functional experiments suggested that EHMT2 was also increasingly expressed in OGD/R-induced BV-2 cells and silencing EHMT2 reduced neuronal damage and restrain inflammatory response. Reportedly, inhibition of EHMT2 conferred neuroprotection in an *in vitro* model of hypoxic metabolic stress [30]. Also, the expression of EHMT2 could be repressed by the sevoflurane treatment, which exerted a protective effect on neurons in hypoxic-ischemic encephalopathy [31]. Furthermore, EHMT2 possesses immunomodulatory effects and is overexpressed in multiple types of cancer. For example, EHMT2 inhibition reduced the expression of pro-inflammatory genes and played an important role in trained immunity in non-muscle-invasive bladder cancer patients [32]. In addition, inhibition of EHMT2 conferred neuroprotection in a model of hypoxic metabolic stress [30]. A previous study demonstrated that sevoflurane post-treatment reduced EHMT2 and H3K9me2 protein levels, increased NRF2 protein levels, alleviated inflammation and neuronal apoptosis, and thus alleviated hypoxic ischemic brain injury (HIBI) in neonatal rats through HIBI models *in vivo* and *in vitro* [16]. Based on these results, it was reasonably concluded that suppression of EHMT2 could relieve neuronal damage and inflammation. Finally, we conducted loss- and gain- of function experiments and found that knockdown of EHMT2 mitigated the neuronal cell damage and the M1 polarization of BV-2 cells caused by overexpressed HMOX1.

In conclusion, down-regulated EHMT2 could depress HMOX1 expression by inhibiting histone methylation, thereby mitigating neuronal damage and blocking cell apoptosis and inflammatory response. While this finding is promising, there are still a number of limitations that need to be overcome before it can be integrated into clinical practice. First, our experiments in this work were carried out at cellular level and it requires more elaborate animal or clinical experiments to validate whether HMOX1 and EHMT2 also have the same effects in ischemic stroke *in vivo*. Next, the number of cell samples used in our experiments was limited. Last, more comprehensive investigations about the regulation of other genes by EHMT2/HMOX1 in ischemic stroke are required in our future study. This regulatory axis EHMT2/HMOX1 may have a potential as a useful diagnostic and therapeutic biomarker in ischemic stroke.

## Supplementary Material

Supplementary Figure
