# Supplementary material for: EHMT2 affects microglia polarization and aggravates neuronal damage and inflammatory response via regulating HMOX1
Source: Transl Neurosci. 2023 Jul 27;14(1):20220276. doi: 10.1515/tnsci-2022-0276 (PMC10388136; doi:10.1515/tnsci-2022-0276)
Supplement: Supplementary Figure [file tnsci-2022-0276-sm.pdf]

Supplementary material

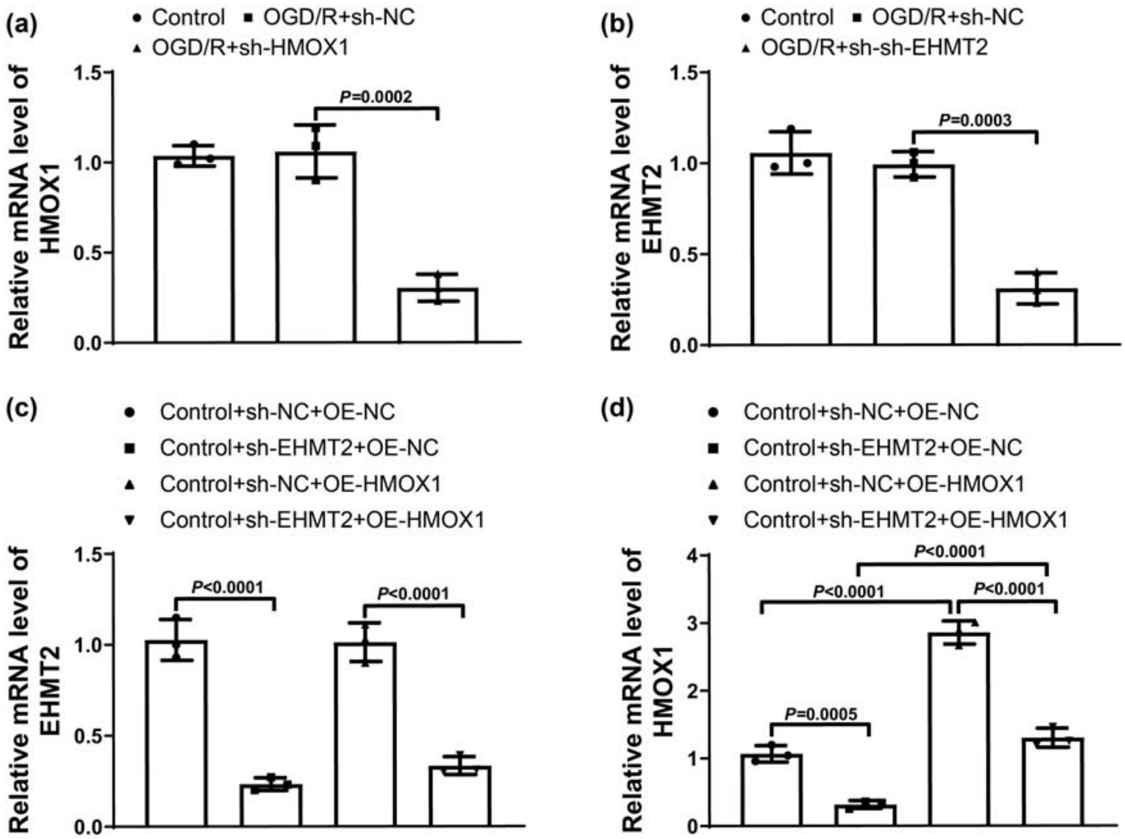

Figure S1: The verification of the transfection efficiency in Control group. Notes: (a to d), qRT-PCR was used to detect the mRNA expression of EHMT2 and HMOX1 in BV-2 cells. Each assay was repeated three times. One-way analysis of variance test was applied for comparisons among multiple groups with Tukey's multiple comparisons test for post hoc tests.
